# Micro and Nano-Scale Technologies for Cell Mechanics

**DOI:** 10.5772/59379

**Published:** 2014-01-01

**Authors:** Mustafa Unal, Yunus Alapan, Hao Jia, Adrienn G. Varga, Keith Angelino, Mahmut Aslan, Ismail Sayin, Chanjuan Han, Yanxia Jiang, Zhehao Zhang, Umut A. Gurkan

**Affiliations:** 1 Department of Electrical Engineering and Computer Science, Case Western Reserve University, Cleveland, USA; 2 Case Biomanufacturing and Microfabrication Laboratory, Case Western Reserve University, Cleveland, USA; 3 Department of Biology, Case Western Reserve University, Cleveland, USA; 4 Department of Biomedical Engineering, Case Western Reserve University, Cleveland, USA; 5 Department of Civil Engineering, Case Western Reserve University, Cleveland, USA; 6 Department of Mechanical and Aerospace Engineering, Case Western Reserve University, Cleveland, USA; 7 Department of Orthopaedics, Case Western Reserve University, Cleveland, USA; 8 Advanced Platform Technology Center, Louis Stokes Cleveland Veterans Affairs Medical Center, Cleveland, USA

**Keywords:** Microfabrication, Nanofabrication, Biophysics, Single Cell Analysis, Mechanical Manipulation, Microfluidics

## Abstract

Cell mechanics is a multidisciplinary field that bridges cell biology, fundamental mechanics, and micro and nanotechnology, which synergize to help us better understand the intricacies and the complex nature of cells in their native environment. With recent advances in nanotechnology, microfabrication methods and micro-electro-mechanical-systems (MEMS), we are now well situated to tap into the complex micro world of cells. The field that brings biology and MEMS together is known as Biological MEMS (BioMEMS). BioMEMS take advantage of systematic design and fabrication methods to create platforms that allow us to study cells like never before. These new technologies have been rapidly advancing the study of cell mechanics. This review article provides a succinct overview of cell mechanics and comprehensively surveys micro and nano-scale technologies that have been specifically developed for and are relevant to the mechanics of cells.

Here we focus on micro and nano-scale technologies, and their applications in biology and medicine, including imaging, single cell analysis, cancer cell mechanics, organ-on-a-chip systems, pathogen detection, implantable devices, neuroscience and neurophysiology. We also provide a perspective on the future directions and challenges of technologies that relate to the mechanics of cells.

## 1. Introduction

Cells, similar to most engineering materials, are subject to different types of physical effects including external forces such as compression, tension, fluid shear stress, hydrostatic pressure and internal forces caused by the cytoskeleton. Cells effectively sense the mechanical cues in their microenvironment and respond accordingly by altering their biological, chemical and physical properties [1]. For example, cells can reinforce their cytoskeleton to create stronger surface adhesion [2] or fluidize their cytoskeleton to decrease their structural stiffness in response to changes in their surroundings [3]. It is well known that biochemical signals are important factors that regulate many cellular processes. Mechanical properties and forces are increasingly being recognized as key players in basic cellular processes, and as part of the extracellular signals that regulate the fate and function of cells [4-9]. Cell mechanics influence a wide range of measures such as morphological changes, migration, proliferation, adhesion and differentiation [10]. These measures take place differently in a state of disease, which is generally due to the altered biochemical as well as mechanical microenvironment [11,12]. The study of cell mechanics is a multidisciplinary field that bridges cell biology with fundamental mechanics, and micro and nanotechnology, which synergize to help us better understand the complex nature of cells in their native environment.

To capture a complete picture of all the essential mechanical interactions and physical properties of cells, we need approaches and technologies, which can work and interact with cells. A typical cell body is about 10 micrometres (μm) in diameter, which is approximately one tenth of the thickness of a human hair. The size or resolution of the tools utilized in cellular biophysical studies has to be in the order of size or smaller. Otherwise, numerous theoretical assumptions would have to be employed and measurement errors may cloud our objective examination. With the recent advances in nanotechnology, microfabrication technologies and micro-electro-mechanical-systems (MEMS), we are now well situated to tap into the wondrous micro world of cells. The field that brings biology and MEMS together is currently known as Biological MEMS (BioMEMS). BioMEMS take advantage of systematic design and fabrication methods to create platforms that allow us to study cells like never before. These new technologies have been rapidly advancing the study of cell mechanics. Therefore, in this review we cover micro and nano-scale technologies that have been specifically developed for and are relevant to the mechanics of cells. We present a comprehensive survey of micro and nano technologies relevant to a wide range of cell types, and their applications in biology and medicine, including imaging, cancer research, neuroscience, tissue engineering, regenerative medicine and pathogen detection.

## 2. Role of cell mechanics in biology and medicine

**Figure 1.**
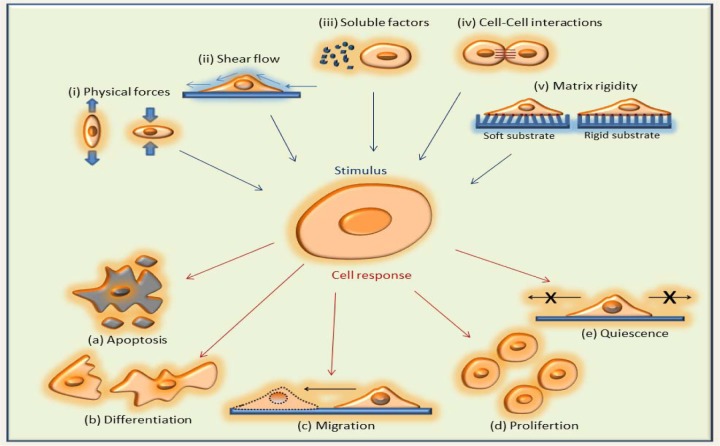
Cells respond to different microenvironmental stimulus *in vivo*. A schematic showing the different factors, (i) physical forces, (ii) shear flow, (iii) soluable factors, (iv) cell-cell interactions and (v) matrix rigity that trigger the cells to undergo changes in their behaviors and functions such as (a) apoptosis, (b) differentiation, (c) migration, (d) proliferation and (e) quiescene.

In general, cells consist of a membrane, cytoplasm, nucleus and a cytoskeleton. The cytoskeleton is composed of a network of filamentous proteins, which include microtubules, intermediate filaments, actin filaments and other cellular proteins [13,14]. Cells have a dynamic nature and undergo different types of intracellular and extracellular events to maintain their essential biological functions (Figure 1), including sensing, cell-cell communication, maturation, migration, proliferation, differentiation, apoptosis and quiescence [15-17]. Cells are extraordinarily amenable to adapting to changes in their physiological microenvironment, which is a complex and ever changing medium that is all around them.

Cells generally respond to the mechanical forces and mechanical properties of their microenvironment in two different ways: with a physical response (e.g., alignment of cell shape and cytoskeleton on anisotropic surfaces) [18-21], or a biochemical response (e.g., activation intracellular or extracellular signaling cascades) [22-24]. These responses lead to the emergence of many cellular events including stiffening, softening, maturation, calcium influx, morphological changes, generation of tractions forces or focal adhesions [25], as well as disease states such as cancer [26,27], osteoporosis [28], osteoarthritis [29], asthma [30], glaucoma [31], malaria [32], atherosclerosis [33] and sickle cell anaemia [34], as a result of complex cellular mechanisms. Therefore, the main objective in studies related to cell mechanics is to understand these interactions, events, and their biological and functional consequences, which are covered in the following sections.

### 2.1 Mechanical properties of cells

The intracellular components of cells such as cytoskeletal proteins, cytoplasm and membrane contribute to the mechanical properties of cells and tissues. For example, the physical properties of the cytoskeleton play an important role in cellular functions such as spreading, crawling, adhesion and polarity. Furthermore, the cytoskeleton is important in maintaining cell shape by providing structural stiffness [13,35,36]. The cell nucleus provides a degree of structural stiffness and plasticity [37,38]. Maintaining the cell shape is crucial to performing biological functions. Cell shape can be determined and controlled by cellular attachments to the surrounding extracellular matrix [39]. The tensegrity (tension integrity) approach states that the combination of tension and compression elements provides a stable form that maintains the cell shape through the balancing of cytoplasmic pressure [14]. According to this approach, cells withstand shape distortion through pre-tension or prestress in their structural elements [14], in which actin filaments and microtubules are the dominant structures for determining cell stiffness [40,41]. Cell stiffness has been extensively studied and quantitative cell stiffness values have been reported in literature (Table 1). Reported cell stiffness values cover a wide range, which mainly depend on the cell type and measurement method. The stiffness of diseased cells can be dramatically different [11,42,43]. For instance, cancer cells are known to be significantly softer than normal cells [11] and sickle red blood cells are known to be significantly stiffer than healthy blood cells [34,43] (Table 1).

**Table 1.** Mechanical properties of cells reported in the literature

Aspects of cell mechanics	Cell type	Magnitude	Tool/Technique	References
Stiffness	Fibroblasts Fibroblasts Vascular endothelial cells Vascular smooth muscle cells Rat ASM(airway smooth muscle)cells	0.02 N/m 0.02 N/m 0.03–0.04 N/m 0.09–0.88 N/m 0.099 N/m	Mcropipette Magnetic twisting cytometer	[231] [232]
Elastic modulus	Cancer MCF-7 cell Osteoblasts Skeletal muscle cells Cardiocytes Erythrocytes Leukocytes Fibroblasts Endothelial cells Outer hair cells	0.95 – 1.19 kPa 0.3–20 kPa 8–45 kPa 90–110 kPa 14–33 kPa 0.2–1.4 kPa 0.6–12 kPa 0.2–2 kPa 2–4 kPa	Atomic force microscopy	[233] [234] [235,236] [237] [238,239] [240] [241,242] [243] [244]
Viscoelasticity	Cytoplasm	210 Pa s 2000 Pa s	Magnetic bead microrheology	[245] [246]
Cell adhesion force	Human cervical carcinoma cell Epithelial cells Murine fibroblast cells Rat cardiac fibroblast	19–204 nN 100 nN 300–400 nN 10 nN	Atomic force microscopy High-speed centrifugation technique Manipulation force microscope Traction force microscopy	[247] [248] [249] [250]
Cell traction forces	Fibroblasts Fish keratocytes	100 nN 20 nN	Microcantilevers micro pads Flexible substrate	[251] [58]
Shear stress	Endothelial cells Bovine aortic endothelial cells Human umbilical vein endothelial cells	1–15 dyn/cm^2^ 10 dyn/cm^2^ 1–3 dyn/cm^2^	Microfluidics	[252-254] [255] [256]

The deformation of certain cell types is indispensable for performing their essential biological functions. For instance, the typical diameter of a red blood cell is about 7.0–8.5 μm and these cells can undergo up to 100% elastic deformation, when they flow through tiny capillaries with inner diameters as little as 3 μm [15]. In sickle cell disease(SCD), due to the intracellular haemoglobin polymerization, red blood cells lose their elasticity. Because of this stiffening, sickled red blood cells cannot deform to pass through capillaries and cause blockages which lead to pain in patients [43]. On the other hand, for some cell types, excessive or repeated mechanical deformation can trigger harmful signaling pathways that can result in physiological disorders or diseases. For example, hypertension and other cardiovascular diseases are associated with an altered compliance of blood vessel walls normally regulated by the deformability of smooth muscle cells [15].

Cells exhibit viscoelastic behaviour which gives them the characteristics of both solids and fluids [44]. Due to their viscoelastic properties, cells deform in a time dependent manner, whereby mechanical stresses relax under constant deformation, or deformation increases over time as a result of a constant load [44,45]. Viscoelasticity plays an important role in cellular processes, such as in the regulation of cell shape and in the regulation of genetic expression through viscoelastic coupling between the plasma membrane and the nucleus [46,47]. The viscoelastic properties of cells have been of interest and they have been studied using the available micro/nano-scale tools (Table 1).

### 2.2 Mechanical interactions of cells

Cells-matrix and cell-cell interactions are involved in numerous signaling pathways and they are required for maintaining the functional and structural integrity of cells. Cells are able to pull and push on their microenvironment through cytoskeletal contractility to sense and assess their microenvironment or other cells [48]. Any miscommunication between cells and the surrounding matrix, or in cell-cell interactions, may lead to the emergence of a disease state [5]. While cell-matrix interactions are primarily mediated by integrins, cell-cell interactions involve the secretion of signaling molecules, gap junctions, neurotransmission, and intercellular nanotubes [5].

Cellular adhesion is a critical process, which governs migration, immobilization and the attachment to the extracellular matrix (ECM). Cellular adhesion is regulated by a combination of several factors including biochemical stimuli, internal and external forces, and the mechanical properties of the extracellular environment [49,50]. For instance, cell adhesion strength increases under mechanical stress, which results in the upregulation of adhesion molecules [48,51]. Furthermore, the presence of mechanical forces can directly impact the size, shape and composition of focal adhesions, implying a direct relationship between the applied forces and the generation of biochemical signals [52]. For example, leucocytes exhibit different adhesive states during an inflammatory response: fast rolling, slow rolling and firm adhesion [53].

In addition to being subjected to external forces, cells can generate their own mechanical forces during migration, contraction and cytoskeletal activity. For instance, all muscle cells have a molecular motor, which is composed of actin and myosin with a well-defined structural arrangement, and used to generate active contraction [54]. Cell traction forces were first observed as distortions on a flexible substrate due to fibroblast locomotion [55]. Traction forces generated by faster migrating cells, such as leukocytes, could not be detected in the same manner as fibroblasts [56], implying that slow moving cells have a stronger adhesion whereas fast moving cells have a weaker adhesion [57]. Therefore, it was determined that slow moving cells have greater cytoskeletal contractility and fast cell migration/motion requires both weak adhesion forces and cytoskeletal contractility [58]. In this sense, traction forces are often measured in slow moving cells, and magnitudes vary depending on the cell type and assessment state (Table 1).

### 2.3 Behaviour of cells in fluid flow

Endothelial cells are the innermost layer in vascular walls and they interact strongly with the blood flow. The essential functions of endothelial cells include the maintenance of the anticoagulant properties of blood vessel walls and the regulation of vascular permeability [59]. When blood flows through the vessels, this generates haemodynamic forces that are essentially a combination of two fluid forces: shear stress and hydrostatic pressure [60]. Even though the entire vascular wall experiences the hydrostatic pressure, only the inner lining endothelial cells undergo blood flow induced shear stress [61,62]. Therefore, the morphology of endothelial cells is affected by shear stress and they align parallel to flow [63,64]. Flow disturbances, separation and vortexes negatively influence the endothelial cell morphology due to cellular mis-orientation [60].

Shear stress on the cell surface leads to intracellular stress generation, cytoskeletal reorganization, and hence the balancing of internal and external forces [59,60,64,65]. Alterations in shear stress may also contribute to vascular diseases as in the example of atherosclerosis [33,66]. Therefore, measurement of the shear stress is an essential aspect of cell mechanics studies. Shear stresses experienced by different cell types cover a wide range as presented in Table 1.

## 3. Conventional analysis and imaging methods for studying cell mechanics

The most commonly used conventional techniques in cell mechanics are: (a) atomic force microscopy (AFM), (b) optical tweezers, (c) micropipettes, (d) flow chambers, and (e) microscopy imaging including confocal and fluorescence microscopy.

### 3.1 Atomic force microscopy

AFM is a type of scanning probe microscopy for imaging, manipulating and quantifying the sample surface at a nano-scale resolution. The basic components of an AFM are a cantilever with a sharp tip controlled by piezoelectric actuators, a laser and a detector. When the tip of the AFM is scanning the sample surface, the cantilever is deflected as a result of the forces between the tip and the surface. This deflection can then be quantified with a detector (photodiodes) by determining the position of the laser beam reflected by the cantilever. AFM has been widely utilized in cell mechanics studies in the literature [67,68]. The cytoskeleton structure was investigated and the mechanical properties including the elasticity, viscoelasticity and plasticity of L929 cells were quantified using AFM force measurements [69]. Elastic modulus and viscosity can be used as indicators of cellular differentiation or can be utilized in observing the effects of external stimuli. Furthermore, AFM can be used as a manipulation tool at the single cell level.

### 3.2 Optical tweezers

Optical tweezers, also known as laser tweezers, have the ability to manipulate dielectric particles by focusing a laser to a diffraction-limited point through a microscope objective that has a high numerical aperture [70]. Particles near the focused laser are trapped because of the restoring force towards the focus. The size of the particles that can be trapped in optical tweezers range from 20 nm to several micrometres, such as organelles, cells and polystyrene or silica microspheres. Furthermore, forces ranging from 0.1 to 100 pN can be exerted using optical tweezers [70]. In a typical study, the mechanical properties of the red blood cell membrane spectrin network were characterized using optical tweezers. The isolated spectrin skeleton was deformed by applying forces to silica beads bound to the membrane [71].

### 3.3 Micropipettes

Micropipettes are used for the mechanical analysis of cells by applying a suction to a small portion of a cell, while measuring the deformation of the cell membrane and the suction pressure of the micropipette [72]. A novel micromanipulation technique was presented to determine alterations in cellular rheology during cell spreading [73]. Chick fibroblasts were allowed to spread on the surface of a glass microplate and micropipette aspiration was applied to cells at controlled pressure levels. The internal pipette radius, cell radius outside the pipette and the length of the aspired portion of the cells were quantified [73]. The automated micropipette aspiration was utilized in conjunction with a video microscopy system. Membrane deformation, membrane area, and cell volume were measured and tracked at a nano-scale resolution.

### 3.4 Flow chambers

Flow chambers simulate fluid shear stresses on cells to mimic their physiological environment. A typical flow chamber includes inlet-outlet ports, a vacuum slot, a gasket that specifies the height of the chamber and a glass coverslip that encloses the chamber, the glass coverslip can be coated with different cell layers or proteins [74]. A mouse endothelial blood-brain barrier (BBB) model including dynamic interactions between T cells and spinal cord microvessels was developed [75]. Flow chambers are widely used in the literature to mimic blood cell-endothelial wall interactions to understand disease pathophysiology such as in SCD. Post-capillary venules, where blood cell-endothelium interactions occur *in vivo* in SCD, were modelled *in vitro* with cultured endothelium on the chamber walls to study abnormal red blood cell adhesion on the endothelium [76,77].

### 3.5 Optical microscopy

Optical microscopy tools have been commonly used in studies of cell mechanics. High resolution imaging and 3D volume construction are invaluable for cell deformation and strain measurements. Modern fluorescent and confocal microscopes offer these properties with live cell imaging functions, which have enabled recent advances in the study of cell mechanics. The confocal microscopy allows point-by-point illumination of the samples using a focused laser beam resulting in higher resolution and 3D information. Fluorescence microscopy is based on obtaining images of fluorophore-labelled samples illuminated with a specific wavelength. Furthermore, a novel confocal microscopy-based indentation system was presented for studying chondrocyte mechanics [78]. 3D reconstructions of the cells were obtained and cellular deformations at different controlled loading conditions were evaluated. A fluorescence microscopy-based 3D particle tracking system was developed for motion tracking within a 100 micrometre range [79]. The viscoelastic mechanical response of kidney cells was analyzed using this technique.

## 4. Micro and nano technologies in cell mechanics

**Figure 2.**
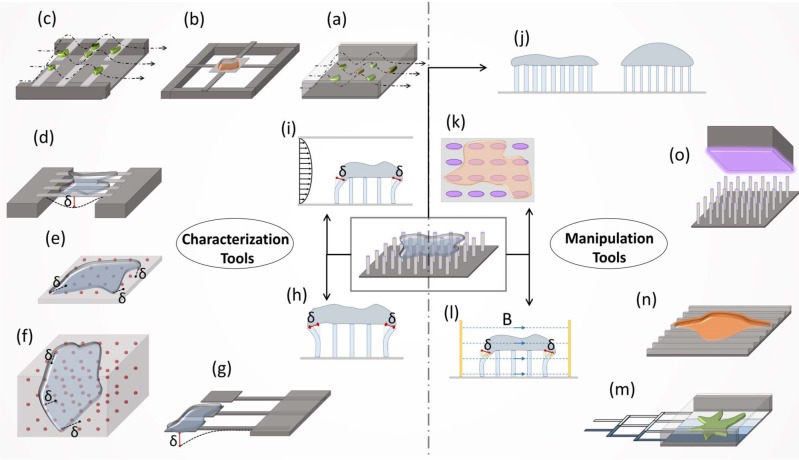
BioMEMS devices in cell mechanics. The tools can be divided into two main categories: characterization tools, for the measurement of the different physical properties of cells, and manipulation tools, for the exertion of an extrinsic effect. (a) The adhesion strength characterization of cells in microfluidic channels is performed by simply counting the cells remaining after shear flow application. (b-c) Measurement of cell mass (b) in microfluidic chip and (c) on pedestals. Both tools are based on the resonance frequency change of the cantilevers or pad after cell attachment. (d) Cellular deformation measurement is performed by using piezoelectric nanoribbons. (e-i) The characterization of traction forces; (e-f) on 2D or in 3D bead embedded gels from the relative displacement of beads on (g) cantilever pads and (h) vertical micropillars is performed by measuring the deflection of cantilevers or micropillars, and (i) on micropillars under shear flow from micropillar displacement. (j-k) The manipulation of the cells by substrate alterations with micropillar configurations of (j) variable stiffness or (k) anisotropic pillar geometry. (l) Deformation application is performed using magnetic nanowires embedded in micropillars in a magnetic field. (m) The generation of substrate gradients is performed via microfluidics. (n) The manipulation of cell shape and phenotype is performed using nanoridge topography. (o) The generation of substrate patterns is performed using microcontact printing. Micropillar and microfluidic based approaches were found to have a variety of applications as both characterization and manipulation tools.

Conventional tools with high sensitivity and accuracy, such as AFM and laser tweezers, have been used extensively for mechanical characterization and the manipulation of cells as described above. While these tools have played an essential role in understanding cell mechanics, they are generally complex, costly and labour-intensive, and they present throughput challenges. Micro/nano tools have been rapidly growing and spreading in the studies of cell mechanics due to their low-cost, easy adaptation and operation, portability, and high-throughput. In this context, MEMS devices for biological studies, which are also known as BioMEMS, provide a great opportunity to study the mechanical aspects of cells (Figure 2).

### 4.1 Measurement of cellular mechanical properties

As discussed in Section 2, cells maintain a biophysical equilibrium with their microenvironment by probing their surroundings in a sensitive and continuous manner. This equilibrium is interrupted by cells in case of any transformational change such as growth, migration, adhesion and differentiation. A biophysical imbalance between a cell and its environment emerges as traction forces, cell deformation and changes in cell mass, which are discussed in the following sections.

#### 4.1.1 Cellular traction

Researchers promoted various methods for measuring traction forces, such as ultrathin silicone films [80,81], and polyacrylamide (PAA) gels cross-linked at different levels [82,83]. The ultrathin film approach measures the amount of traction force by examining the wrinkling of the film by the cells. Even though this method provided an important insight in earlier studies in the 1980s and 90s, measuring forces from wrinkles is complicated [84]. On the other hand, fluorescent microbead embedded PAA gels provide a more accurate quantification of the traction forces (Figure 2e). For example, Dembo et al. [82] studied the traction forces at focal adhesions during the locomotion of single 3T3 fibroblast cells using collagen conjugated polyacrylamide gels with embedded fluorescent marker beads. Moreover, gel stiffness can be tuned by changing the cross-linking level. However, changing the cross-linking level not only alters the mechanical properties of the substrate, but also has an effect on the porosity, surface chemistry and binding properties of the ligands [85]. Thus, this process makes it hard to isolate the effects of one substrate property change from others.

In the last decade, other methods based on micro/nano cantilevers or pillars have been used extensively to study traction forces (Figure 2g-l) [86,87]. When the cells are cultured on top of functionalized (adding ECM proteins) pillar arrays, they form focal adhesions with the pillar top surface and apply traction forces through these adhesion points. Under these traction forces, pillars behave like simple springs, which translate into forces that are linearly correlated with the deflections of the pillars. Thus, by measuring these deflections, the traction forces of the cells can be calculated.

Pioneers of this approach measured the traction forces of fibroblast cells during migration by utilizing flexible horizontal cantilevers [88]. The BioMEMS device used in the study incorporated mounted horizontal cantilevers and pads, whereby cell-surface interactions occur at the tip of these cantilevers. By imaging the deflection of these cantilevers, they were able to calculate traction forces (Figure 2g). Even though this method overcomes the computational and material complexities of the bead embedded gel approach, it is limited to forces generated at only one direction and location.

Tan et al. [86] investigated the interaction between cells and their substrates by seeding cells on micro pillars of 3 μm in diameter and 11 μm in height (Figure 2h). From the deflection of the pillars, they determined the traction forces applied by the cells and correlated these traction forces with the distribution of the focal adhesion on each post. They found that there were two groups of adhesions causing the traction forces. Forces generated by the first group increased with an adhesion size greater than 1 μm^2^, whereas there were no such correlations for adhesion sizes less than 1 μm^2^.

Rabodzey et al. [89] investigated the shear forces induced at cell-cell junctions during the neutrophil transmigration of vascular endothelium by growing endothelium on micro pillars (2 μm in diameter and 3.3 μm to 4.7 μm in height) in an *in vitro* laminar flow chamber (Figure 2i). They showed an increase in traction force during the intercellular penetration of neutrophils and gap formation. There was also an increase in traction forces applied by endothelial cells in response to the penetration and destruction of cell junctions. Based on these results, they suggested that a successful transmigration of a neutrophil through the endothelial monolayer depends on the competition between the cell-cell junctions and the cell-substrate.

Even though micropillars have great advantages due to their inherently simple structure, there are some limitations associated with them. For example, the nontrivial topology of the micropillars might affect cell adhesion for certain geometrical configurations. Moreover, some cell types require extra soft substrate stiffness which can be hard to achieve using micropillars due to manufacturing requirements [84]. Furthermore, both micropillar and gel approaches employ two dimensional (2D) substrates, which can cause cells to behave in a different way than in their native three dimensional (3D) environment.

In a recent study, Legant et al. [90] investigated the traction forces of EGFP-expressing 3T3 fibroblast cells in 3D elastic hydrogel matrices by exploiting the relative deformation of embedded fluorescent beads (Figure 2f). Even though this study employed a 3D environment for the cells, this approach is still susceptible to computationally intensive data processing. In a different approach, Marelli et al. [91] fabricated flexible curved cantilevers that suspend cells in 3D to measure traction forces. A limitation of this method is the restriction of cells in a confined configuration, which prevents cell migration and cell-cell interactions.

#### 4.1.2 Cellular deformation

Cell deformation can be a significant indicator of the various vital functions of cells such as growth, locomotion and depolarization. Nguyen et al. [92] developed a new method to measure the mechanical responses of cells to electrical stimulations. The authors fabricated piezoelectric PbZrxTi1-xO3 (PZT) nanoribbons to measure the deformation of neuronal cells undergoing electrical excitations. The results showed a 1 nm cell deformation in response to a 120 mV stimulus. This result was in agreement with a theoretical model of a depolarized cell membrane experiencing tension (Figure 2d).

#### 4.1.3 Cell mass

Cell mass can be used as an indicator of protein synthesis, DNA replication and other large molecule accumulation inside the cell during growth and differentiation [93]. Researchers have developed several approaches to measure cell mass incorporating both micro structures and microfluidics (Figure 2c).

Park et al. [93] developed a BioMEMS device that involves a microfluidic channel and horizontal cantilever arrays mounted on the sidewalls of the channel (Figure 2c). In this study, HeLa cells were injected into the channel and captured on the cantilevers by using positive dielectrophoresis. After culturing adhered cells on cantilevers for a period of time, the resonance frequency of cantilevers was measured with a Laser Doppler Vibrometer. Since the resonance frequency is correlated with the spring constant and the mass of the system, cell mass was calculated from the resonance frequency shift of the cantilevers.

Grover et al. [94] also used microfluidics, cantilevers and resonance frequency to measure cell mass using a different approach. The authors developed a microfluidic chip with two different fluid flows at the lateral sides and incorporated a cantilever at the location where the two fluids mixed. When a cell was flowing with the first fluid and passed through the cantilever, the resonance frequency was measured. Next, the fluid flow was reversed and the cell passed through the cantilever a second time, and the resonance frequency was measured again. The resonance frequency of the cantilever was proportional to the cell's buoyant mass in the fluid when the cell passed by the cantilever. From these measurements, absolute density, mass and the volume of a cell can be calculated according to Archimedes' law.

#### 4.1.4 Cellular adhesion

The adhesion strength of cells can be quantified and used for practical purpose. Singh et al. [95] developed a microfluidic chip to measure cell adhesion strength and, then, isolate stem cells based on their specific adhesion strengths. In the microfluidic chip (Figure 2a), cells were subjected constantly to a fluid flow that could detach the cells from the surface. The authors showed significant difference in the adhesion strength between somatic, pluripotent, partially programmed and differentiated progeny cells. They used this variation in adhesion strength to isolate specific cell populations with 95%–99% purity and >80% survival.

### 4.2 Mechanical manipulation of cells

Initial studies on cell mechanics focused on the mechanical characterization of cells. Later, it was realized that the field required micro and nano instruments that can manipulate and simulate the mechanical environment of cells to further investigate cell mechanobiology. The properties and characteristics of some of these methods are reviewed in this section.

#### 4.2.1 Magnetic pillars

Using a micropillar array (Figure 2l), Sniadecki et al. [96] applied an external magnetic field force to adhere cells to micropillars, some of which had embedded magnetic nanowires. It was reported that applying a magnetic force, which deflects the magnetic pillars, increases the focal adhesion size only at these pillars, but not at the nearby nonmagnetic pillars. The results showed that applying such a force caused a loss in contractility at discrete locations of the cell's periphery.

#### 4.2.2 Shear flow

Ting et al. [97] investigated the effects of fluid shear stress on the cytoskeleton and cell-cell contacts of endothelial cells. In this study, endothelial monolayers were grown on two different micropillar arrays in a flow chamber. One of the micropillar arrays was placed close to the inlet and the other one was positioned around the middle of the chamber, to achieve both disturbed and laminar flow conditions, respectively. It was reported that while laminar flow conditions increased cytoskeletal tension, disturbed flow had a reverse effect.

#### 4.2.3 Protein micropatterning

Protein micropatterning has been used to control and manipulate cell geometry, traction, migration and adhesion. To pattern proteins on a substrate, microfluidic and microcontact printing methods have been used (Figure 2m-o). In microfluidic patterning, proteins are immobilized on a substrate placed inside a microfluidic channel in which gradients are obtained by using a series of serpentine channels mixing different solutions at different ratios (Figure 2m). Dertinger et al. [98] generated laminin gradients to study axonal specification in neuronal cells. In another study, Rhoads et al. [99] investigated fibroblast haptotaxis using fibronectin gradients produced via microfluidics. A challenge of microfluidic patterning is that the patterned substrate remains in a closed system after the pattering is completed.

Another method of protein microcontact printing works by absorbing the protein of interest on a stamp and putting this stamp in conformal contact with a microengineered substrate (Figure 2o). Tan et al. [86] functionalized the top surface of micropillar arrays with ECM proteins by using microcontact printing. Protein functionalization was applied either to all of the pillar arrays or to a constrained smaller area. This approach allowed cell behaviour and traction forces to be analyzed in a restricted area.

#### 4.2.4 Topographic modification

Topographical cues on a surface are crucial for cell morphology, migration and differentiation. Therefore, Teixeira et al. [100] investigated the morphological and biophysical behaviour of human corneal epithelial cells on substrates with nano grooves and ridges (Figure 2n). The study utilized topographically patterned substrates with feature dimensions ranging from as small as 70 nm to 2.1 μm, feature pitch between 400 nm to 4 μm, and groove depths of 150 nm and 600 nm. It was reported that epithelial cells elongated and aligned on substrates with ridge and groove features, whereas smooth surfaces caused the cells to assume round morphologies.

Bucaro et al. [101] investigated the relationship between the geometry of nanopillars (spacing and aspect ratio) and stem cell morphology. They used nanopillar arrays ranging from 0.2 to 0.5 μm in diameter, 5 μm to 10 μm in height, and 0.8 μm to 5μm in spacing. Based on the findings, they proposed a critical spacing distance at which the extensions of cells could only grow in the direction where the inter-pillar distances were the shortest. On the other hand, sub-critical spacing led cells to spread radially as focal adhesions could be established in every direction. However, cells showed no bridging over the nano pillars when over the critical spacing distance. Instead they spread at the base of the nano pillars with increased branching of the extensions. Moreover, a dramatic increase in cell polarization and alignment were observed with the increase in pillar aspect ratio (thus a reduction in the bending stiffness of the pillars).

#### 4.2.5 Substrate stiffness

Saez et al. [102] fabricated elliptical micropillars to obtain anisotropic stiffness characteristics in each pillar to study the directional epithelial growth and the migration of epithelial cells. It was observed that cells migrated in the direction of the greatest stiffness (Fig 2k). Fu et al. [85] investigated the effect of substrate rigidity on cell morphology, focal adhesion, cytoskeletal contractility and stem cell differentiation by using micropillar arrays with different stiffness values (Fig 2j). In this study, stiffness was controlled by varying the pillar height. Results showed that cells displayed spherical morphology at lower stiffness arrays, whereas they displayed spreading on more rigid arrays. MSCs cultured on rigid substrates tended to an osteogenic fate, whereas on soft micropillar arrays, they favoured an adipogenic fate.

## 5. Emerging areas of application in biology and medicine

Micro and nano technologies are used in a broad range of applications, including single cell analysis, cancer cell mechanics, organ-on-a-chip systems, pathogen detection, implantable devices, and neurobiology. These emerging applications are reviewed in this section.

### 5.1 Single cell analysis

Single cell isolation is a micro-scale mechanical technique crucial for understanding processes at a cellular level. With single cell analysis, important variations in a cellular population can be detected and analyzed, which is not possible in a bulk analysis. It opens new dimensions for the study of rare cells, such as stem cells, circulating tumour cells, and biological samples collected from patients. The mechanical trapping of single cells in microfluidic channels include lateral and planar trapping using side structures or U shaped micro-apertures or microwells located under the cells [103-110]. Another way to trap single cells is to employ pneumatic valves integrated in microfluidic systems [111]. Nanolitre volumes of reagents can be applied to the isolated cells by using integrated valves and pumps. Several assays based on this system were developed including cell viability. These include assay and ionophore-mediated intracellular Ca2+ flux measurements, and multistep receptor-mediated Ca2+ measurements.

A third method to trap single cells is droplet encapsulation, in which individual cells can be isolated in extremely small volumes [112]. After capturing the cells inside droplets, they can be easily used in various assays, including, cytotoxicity screening and viability [113], isolation and protein/DNA purification [114-116], therapeutic applications [117] and subcellular organelle studies [118].

The biochemical analysis of single cells has been considered one of the holy grails in cell biology and has become possible with microfluidic technology. There are different protocols for dissolving the cellular membrane to access the intracellular contents. Chemical lysis, using detergents, using alkaline conditions, electrical lysis, laser lyses, mechanical lysis and thermal lysis can be used for lysing the cellular membrane [119-125].

Important developments have been made in microfluidic systems and these have been integrated with major analytical methods currently used for genomics and proteomics for single cell analysis. The gene expression analysis of single cells has been demonstrated in various studies such as the quantification of mRNA from two distinct populations [126] and the combination of microfluidic systems with qRT-PCR [127]. Microfluidic systems can be used to study single cell proteomics. A challenge in single cell proteomics is the amount of protein that can be isolated from an individual cell. The concentration of some proteins in a cell may be extremely low, and they cannot be amplified as in DNA and RNA isolation. Therefore, sensitivity is essential in single cell proteomics. In such a system, a microfluidic approach was used to manipulate, lyse, label, separate, and quantify the b2 adrenergic receptor contents of a single cell using single-molecule fluorescence counting [128]. As another approach, a microfluidic system incorporated with a mass spectroscopy was used to analyze proteins in a single cell [129].

### 5.2 Cancer cell mechanics

Cancer cell mechanics provides a promising opportunity to understand how cancer cells malignantly grow, transform, aggressively spread and invade normal tissues. Cancer cells are known as malfunctioning biological cells in the human body, which can uncontrollably proliferate and disrupt the organization of tissue [26]. Cancer cells tend to adapt themselves to squeeze and spread in normal tissues or blood vessels. Therefore, they deform more easily than normal cells, which was observed in many studies using breast cancer cells [12,67,130], hepatoma cells [131,132], and HeLa cells [133,134]. Such differences in mechanical properties may be regarded as an inherent marker for cancer diagnosis and treatment. On the other hand, cancer cells may respond and behave differently to external stimulations (extracellular matrices and induced forces). For example, cancer cells were found to exhibit a larger traction force than normal cells by approximately 20% for HeLa cells and 50% for L929 cells on micropatterned substrates [134]. Cancer cell behaviours such as adhesion, migration and division were studied under different mechanically and magnetically induced stimulations [135-137], which help us understand metastatic mechanisms.

Currently, the integration of MEMS and imaging techniques [133,136-140] are inspiring more interest in exploring how cancer cells respond to ECM and external forces. The conversion of these mechanical signals into chemical signals results in adaptive changes in cellular behaviours, such as cell adhesion, migration and division.

BioMEMS devices, to date, have played a dominant role in the studies of cancer cell mechanics due to the following reasons: (1) BioMEMS devices provide a platform which can better mimic the *in vivo* environment. For example, micropatterned matrices [133] for studying cell exerted traction forces and migration can better mimic the microenvironment of cancer cells. (2) BioMEMS devices exhibit higher precisely-controlled and spatially-resolved forces. Micropost arrays [86,134,141] and microfluidic assays [142] can precisely control forces by changing geometry or fluid velocity. (3) BioMEMS devices enable the analysis of cancer cells with higher accuracy and throughput compared to conventional tools.

**Figure 3.**
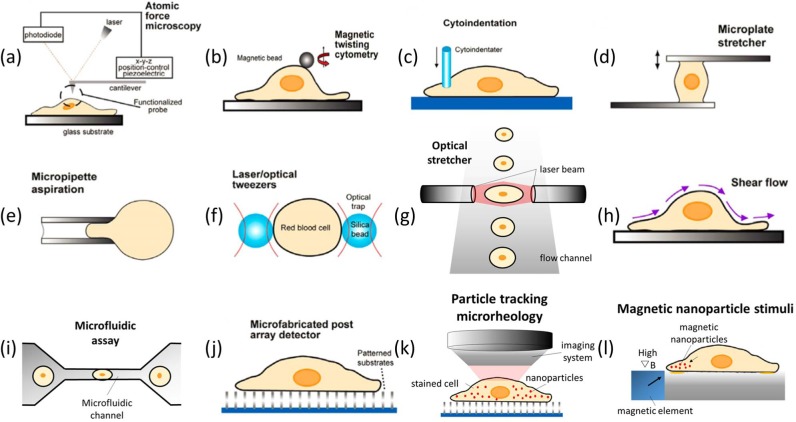
Major techniques for cancer cell mechanics study. (a) Atomic force spectroscopy; (b) magnetic twisting cytometry; (c) cytoindentation; (d) microplate stretcher; (e) micropipette aspiration; (f) laser/optical tweezers; (g) optical stretcher; (h) shear flow; (i) microfluidic assay; (j) microfabricated post array; (k) particle tracking microrheology; (l) magnetic nanoparticle-based stimuli. This figure [26] is reused with permission from Elsevier.

Techniques to study cancer cell mechanics can be categorized in terms of their applications in cancer cell mechanics and cell-ECM interactions (Figure 3). Principles, advantages, limitations and applications are summarized in Table 2. AFM-based methods (Figure 3a) [67,68], magnetic twisting cytometry (Figure 3b) [131,143] and cytoindentation (Figure 3c) [12,144] usually exert local forces on a cell. Therefore, they suffer from low throughput and direct contact with the cell surface may cause active cellular responses. Single-cell-based techniques such as microplate stretcher (Figure 3d) [145,146] and micropipette aspiration (Figure 3e) [132,147,148] have modest throughput, but they also cannot avoid cell-tool interactions either. Optical techniques such as optical tweezers (Figure 3f) [149-152] and optical stretchers (Figure 3g) [153-155] minimize active cell responses when deforming cancer cells without contact. The optical stretcher takes advantage of a microfluidic channel to mimic the *in vivo* environment and uses two counter-propagating divergent laser beams to suspend and deform cancer cells. The laser induced forces can be precisely controlled by output power. For example, using this technique, Guck et al. found that the deformability of SV-T2 cells was significantly higher compared to BALB/3T3 cells [155].

**Table 2.** Methods for studying cancer cells mechanics and mechanical properties of cancer cells reported in the literature

Methods	Induced force		Advantages	Disadvantages	Applications				References
					Case study	Major observations	Typical values		
							Cancer cells	Normal cells	
Atomic force microscopy (AFM)	Partial of cell	10^−7^–10^−11^N	1) High resolution; 2) Low forces with minimal disruption; 3) Three dimensional surface profile.	1) Can be used only for cells that adhered to a substrate; 2) Relatively slow scanning rate; 3)Mechanical contact may lead to cellular response.	Stiffness of metastatic cancer cells from lung, breast and pancreas [67]	Stiffness of metastatic cancer cells is more than 70% softer than the benign cells	Lung: 0.56±0.09kPa; Breast: 0.50±0.08kPa; Pancreas: 0.54±0.08kPa;	Lung: 2.10±0.79kPa; Breast: 1.93±0.50kPa; Pancreas: 0.54±0.12kPa;	[67,68,257]
					Stiffness and adhesion forces of metastatic cancer cells and benign mesothelial cells [68]	Metastatic tumour cells are more than 80% softer than benign cells and surface adhesion is ∼33% less than normal cells.	Stiffness: 0.38±0.20kPa; Adhesion force: 34.2±5.3pN.	Stiffness: 2.53±1.30kPa; Adhesion force: 51.1±15.2pN.	
Magnetic twisting cytometry (MTC)	Partial of cell	10^−10^–10^−12^N	Probe the single cell with very small deformations and over wide ranges of time scale and amplitude.	1) The bead localization on the cell is random; 2) The induced bead rotation and displacement are strongly dependent on the bead attachment angle.	The effects of tubeimoside I (TBMS I) on human hepatoma (HepG2) cells[131]	The stiffness of HepG2 cells decreased consistently with the increased concentration of TBMS I exposure. In addition, the HepG2 cells responded to TBMS I much faster than the normal liver (L-02) cells.	Stiffness (HepG2): 0.44±0.01 Pa/nm; Respond time: 73s.	Stiffness(L-02): 0.88±0.04 Pa/nm, Respond time: 109s.	[131,143,257]
Cytoindentation	Partial of cell	10^−7^–10^−9^N	1) Simple modelling of the viscoelastic behaviour of the cells; 2) Detection of differences in cell mechanics; 3) The probe is large to measure bulk cellular properties.	The response may depend significantly on the precise probing location.	The elasticity of benign (MCF-10A) and cancerous (MCF-7) human breast epithelial cells [12]	Apparent Young's modulus of malignant (MCF-7) cells significantly decreased, (1.4–1.8 times) than that of non-malignant (MCF-10A) cells at physiological temperature (37°C), and their apparent Young's modulus increased with loading rate.	–	–	[12,144,257]
Microplate stretcher	Single cell	10^−7^–10^−9^N	Sufficient to induce significant deformation of an entire cell.	1) Time consuming; 2) Low throughput; 3) Can only be applied to extremely well adhering cells.	Elastic response and energy dissipation under repeated tensile loading of epithelial pancreatic cancer cells [146]	The elastic modulus of Panc-1 pancreatic cancer cells decreased after treatment with SPC.	Before SPC: 28.8 ± 2.6 mN m^−1^. After SPC: 16.3 ± 1.1 mN m^−1^.	–	[145,146,257]
Micropipette aspiration(MA)	Single cell	10^−7^N- 10^−10^N	1) Allows for real time correlation of pressure and whole cell deformation; 2) High accuracy; 3) Aspiration pressure can be maintained over a specified duration.	Analytical or computational models are often necessary to derive material properties and the underlying assumptions may at times be difficult to validate.	Viscoelastic properties of human hepatocytes and hepatocellular carcinoma (HCC) cells[132]	HCC cells have higherelastic coefficients but not viscous coefficients compared to than hepatocytes.	Hepatocellular carcinoma (HCC): K1=103.6±12.6N.m^−2^; K2=42.5±10.4N.m^−2^; μ=4.5±1.9Pa.s.	Hepatocytes: Kl=87.5±12.1N.m^−2^; K2=33.3±10.3N.m^−2^; μ=5.9±3.0Pa.s.	[132,147,148, 156,257]
Laser/optical tweezers(OT)	Single cell	10^−11^–10^−14^N	A focused laser beam allows precise bead manipulation in all directions.	1) Force level is limited to induce larger deformation; 2) Larger force would require higher laser power that could excessively heat the cell.	Elasticity of myeloblasts (62–71 CD33^+^CD34^+^cells and 57–63 CD33^+^CD34-cells) from AML patients[150]	The induced deformation of CD33^+^CD^+^cells is greater than CD33^+^CD34-cells under the same stretching force.	The elastic area compressibility modulus, kα= 1.40±0.71 N/m (CD33+CD34-).	The elastic area compressibility modulus, kα= 0.25±0.15 N/m (CD33+CD34+).	[168–171, 267]
Optical stretcher	Single cell	10^−9^–10−^11^N	1) Cells can be suspended to eliminate mechanical contact; 2) Very small numbers of cells are required for distinction; 3) Relatively high throughput using a microfluidic channel.	1) Laser power should be controlled without damaging the cells; 2) Limitations may exist when probing stiffer cells.	Optical deformation (OD) of mouse fibroblasts and human breast epithelial cells [155]	Optical deformability of the SV-T2 cells is significantly increased compared to the BALB/3T3 cells; the cancerous MCF-7 cells are deformed more than the normal MCF-10 cells, and the metastatic modMCF-7 are deformed even more than the nonmetastatic MCF-7.	OD_SV-T2_ = 11.7±1.1 OD_MCF-7_ = 21.4±1.1; ODmodMCF-7 = 30.4±1.8.	OD_BALB/3T3_ = 8.4±1.0; OD_MCF-10_ = 10.5±0.8.	[153-156,257]
Shear flow	Cell populations	1–100Pa	Cone and plate rheometers allow precise control over the applied shear stress.	1) Difficult to visualize induced cellular deformations; 2) Small variations in the cell height and topology can cause local variations of shear stress.	Influence of shear flow on the adhesion of nonmetastatic (MCF-7) and highly metastatic (MDA-MB-435) cells[135]	Detachment of the nonmetastatic MCF-7 cell line decreased significantly while detachment of the highly metastatic MDA-MB-435 significantly increased after 15 hour exposure of a 15 dyn/cm^2^ shear stress.	Detachment (MCF-7) decreased from 44.0±4.6% to 12.1±3.7%; Detachment (MDA-MB-435) ncreased from 37.2±6.3% to 36.2±2.1%.	–	[135,257,258]
Microfluidic assay	Cell populations	–	1) High throughput; 2) Can mimic *in vivo* environment; 3) High accuracy; 4) Easy to fabricate and low cost.	Microfluidic channels need to be properly designed.	Deformability of benign breast epithelial cells (MCF-10A) and nonmetastatic tumour breast cells (MCF-7) [130].	Transit velocity is not significantly affected by cell type. MCF-10A cells were found to have longer entry time than MCF-7 cells of similar sizes, MCF-10A is stiffer than MCF-7 cells.	MCF-10A intry time: 1.698±0.201s; ilongation index: 1.231±0.01191; Transit velocity: 187.0±7.920μm/s.	MCF-7 Entry time: 0.433±0.045s; Elongation index 1.281±0.01505; Transit velocity: 177.3±9.836 μm/s.	[130,142,259]
Microfabricated post array	Single cell/Cell populations	10^−7^–10^−9^N	1) Force application is localized and can be measured with high resolution; 2) The geometry and stiffness of micropillars can be independently adjusted; 3)Can be applied for cell adhesion force, traction force and migration studies.	1) Substrate has a nontrivial topology that might affect cell adhesion and bias the measurements; 2) Substrate may cause active cell-substrate response.	Traction forces exerted by cancer cells [134]	Cancer cell exhibits a larger traction force than the normal cell by ∼20% for a HeLa cell and ∼50% for a L929 cell.	Traction forces of Hela cell: 2.84±0.49 *μN* and L929 cell: 3.48±0.46 *μN.*	Traction forces: 2.3210.16μN.	[86,134,141,257]
Nanoparticle-based techniques	Cell populations	–	It can provide insight into intracellular dynamics and structure, as well as into active transport processes.	1) Hard transferring of experimental observations to theoretical and phenomenological models; 2) Nonlinear effects.	ECM stiffness on the intracellular rheology of cancer cells [33].	In 3D matrices, the intracellular effective creep compliance of prostate cancer cells is shown to increase with increasing ECM stiffness, whereas modulating ECM stiffness does not significantly affect the intracellular mechanical state when cells are attached to 2D matrices.	–	–	[133,136-140,257]
	Partial of cell/Cell population	10^−7^ −10^−9^N	1) High spatially and resolved force with long duration and repeats. 2) Localized stimuli and cell population measurement are both achieved, which increases the throughput.	1) System is complex, which limits its real application; 2) Both cells and magnetic elements are needed to be patterned, and the alignment between them should be precisely controlled.	The influence of mechanical forces on single-cell behaviour [137].	Nanoparticle-induced tension generates filopodia asymmetry and bias metaphase-plate orientation of Hela cells.	–	–	

Microfluidic techniques provide versatility in cancer cell mechanics under precisely controlled fluid flow. For example, using the shear flow technique (Figure 3h), Moss et al. [135] found that the detachment of the nonmetastatic MCF-7 cell line decreased significantly while the detachment of the highly metastatic MDA-MB-435 significantly increased after a 15 hour exposure of a 15 dyn/cm^2^ shear stress. In another example, Tan et al. [86] used microfluidic assays (Figure 3i) as a “deformation passage” for the cells to pass through, and MCF-10A cells were found to have a longer entry time than MCF-7 cells of similar sizes indicating that MCF-10A was stiffer than MCF-7 cells.

Cell traction force is important for many biological processes, such as mechanical signal transmission and cell migration. Therefore, measuring cells' exerted traction forces may provide a better understanding of cancer cell metastasis [154,156]. The micropost array technique (Figure 3j) has been previously used to measure the traction forces of cancer cells [86,134,141]. Similar to the micropost array, Li et al. [134] presented a silicon-nanowire-array-based technique for quantifying the traction forces of three distinct groups of cells: normal mammalian cells, benign cells (L929) and malignant cells (HeLa). The results indicated that cancer cells exhibited a larger traction force than normal cells [134].

To study the interactions between the mechanical properties of ECM and those of the cancer cells, Baker et al. [133,136,138-140] developed an innovative fluorescent nanoparticle-based tracking microrheology method (Figure 3k) [136]. They showed that the intracellular effective creep compliance of prostate cancer cells increased with increasing ECM stiffness in 3D matrices, whereas modulating ECM stiffness did not significantly affect the intracellular mechanical state when the cells are adhered to 2D matrices [136].

Magnetic nanoparticle induced stimuli (Figure 3l) [137] is an innovative approach whereby magnetic fields are used to exert highly localized and spatially resolved forces on the cell membrane. Using this technique, Tseng et al. [137] observed that magnetic nanoparticle induced tension could generate asymmetrical filopodia. Furthermore, as particle-applied forces increased, filopodia protrusions appeared more frequently, emanating from the region to which the forces were applied.

### 5.3 Organ-on-a-chip systems

Cell culture technologies have a broad range of applications in cell and molecular biology research, tissue engineering and drug screening assays. The traditional, yet-still-prevalent 2D cell culture typically seeds cells on the surface of plastic flasks, petri dishes or well plates, where a cell monolayer is formed within the bulk culturing medium. Despite the 2D nature of these systems, numerous biological studies have been performed based on this platform [157-160]. 2D cell cultures have certain limitations: the cell culture conditions poorly mimic the cellular environment *in vivo*, soluble growth factors can be present at abnormally high concentrations, 3D cues are largely absent, oxygen tension can be too high, and cell–cell interactions are rarely seen [161].

The pursuit of gaining a better understanding of the effect of living tissue environment on cells, cell-cell interactions and the response of cells in a natural environment has led to the wave of research efforts aiming to build more natural conditions, termed 3D cell cultures. Leveraging microfabrication techniques such as soft lithography, microfluidics and micropatterning, superior 3D cell culture systems have been developed [162-166]. These techniques can create more natural environments by mimicking 3D ECM structures, applying microfluidic networks capable of transporting nutrients and oxygen, and exerting mechanical loads on cells. The study of the seeding, stimulating and proliferating of cells under these conditions has provided a more accurate understanding of cell mechanics and the effect of environmental cues [167-170].

Microfabrication has enabled co-cultures of various cell types in a single platform with physiological environmental conditions. These platforms are called organ-on-a-chip devices when these sytems are specialized to mimic the function of a specific organ. The development of these microengineering approaches has opened up new possibilities for creating *in vitro* models that reconstitute more complex 3D organ-level structures and for integrating crucial dynamic mechanical cues as well as chemical signals [166,170,171].

There have been encouraging breakthroughs in devising and constructing devices that resemble the structure of the human lungs [172-174], liver [175-178], intestines [164,179,180], kidneys [181,182], cancer tissue [183] and artificial cells in regenerative medicine [117,166,170]. These devices are able to perform either specific healthy physiology functions or pathological conditions of human organs. As an example, the human lung-on-a-chip system was used to show that lung tissue responds differently to bacteria and inflammatory cytokines in the presence of a cyclic mechanical strain [172]. Mechanical cues were shown to accentuate the toxic and inflammatory responses of the lungs to silica nanoparticles, as similar effects were observed in a whole mouse model, proving the system's potential for drug screening and toxicology applications [172].

Recently, a multi-organ-chip with co-cultures of 3D human artificial liver and skin tissues has been designed and tested [184]. The system was shown to support two different culture modes, with tissue exposed to fluid flow or tissue shielded by standard cultures from the underlying fluid flow. This system has provided long-term cultures over 28 days, while supporting tissue crosstalk.

The development of microfabrication technologies to maintain cells in their 3D natural environments *in vitro* and construct higher organ-level microsystems is still in its primitive stages. Researchers are still exploring how to optimize the properties of culturing systems by exploiting more biocompatible materials that better mimic ECMs, to improve cell viability, to form organized cellular structures and to integrate more functions into a single platform. Micro and nano technologies hold enormous possibilities, and they provide powerful and promising approaches for the next generation of organon-chip platforms.

### 5.4 Pathogen detection based on physical properties

Pathogens, including bacteria, viruses, microbes and other microorganisms, are associated with many different fields of research, including diagnostics, pathology, drug discovery, clinical research, biological warfare, disease outbreaks and food safety [185]. Conventional pathogen detection methods involve cell culture, polymerase chain reaction (PCR)-based methods and enzyme-linked immunosorbent assays (ELISA) [186]. Despite the high accuracy and sensitivity, conventional pathogen detection methods are time-consuming due to the complex procedures involved. Recent advances in BioMEMS techniques provide new opportunities to develop biosensors for pathogen detection with simpler processes and smaller dimensions [187-190]. A biosensor is an analytical device that combines a sensitive bioreceptor element with a biological signal transducer to detect an analyte [191]. A bioreceptor (e.g., cell, microorganism, enzyme, antibody, nucleic acid) is used for interactions with the analyte, while a biological signal transducer can convert a signal introduced by the interaction of the analyte with a biological element into another signal that can be more easily measured and quantified. Generally, transduction principles can be classified into three primary categories: optical, electrochemical, and mechanical.

Optical-based biosensors share the advantages of high sensitivity, flexibility and resistance to electrical noise [192]. The most popular optical-based biosensors can be divided into three types: surface plasmon resonance (SPR), chemiluminescence and fluorescence. Due to the advantages of label-free, high sensitivity and real-time measurements, SPR biosensors have attracted much attention recently, and many commercial biosensors based on SPR have been employed in a range of applications, from fundamental studies to clinical diagnosis [193].

Chemiluminescence is a kind of light generated during a chemical reaction, containing electrochemiluminescence (where the luminescence comes from the electrochemical reaction) and bioluminescence (where the emission is produced by living organisms). Chemiluminescence-based methods are superior to other optical methods, because the absence of an external light source not only simplifies the method, but also reduces the detection noise [194]. Paper-based chemiluminescence ELISA is capable of achieving a good sensitivity and linear range for different antigens, which is needed in clinical applications [195]. Based on the chemiluminescence technique, Wolter et al. [196] developed the first flow-through chemiluminescence microarray. This semi-automated readout system provided rapid and simultaneous detections of Escherichia coli, Salmonella typhimurium and Legionella pneumophila within 13 mins, with detection limits of 105, 3×103 and 3×106 cells/mL. A chemiluminescence (CL) flow-through DNA microarray assay was later reported by the same research group. By introducing the stop-PCR method, this system achieved a lower detection limit of 10–100 cells/mL compared to an antibody microarray [197].

Fluorescence is the dominant optical detection method for pathogen detection, which is based on the relaxation process whereby the excited electron returns to its ground state [194]. In addition to its general merits, such as high sensitivity and easy incorporation into microfluidic devices, fluorescence detection displays a distinct advantage in terms of its detection limit for low signal cross-talk from other species, because only structurally rigid compounds with unsaturated or aromatic functional groups can emit fluorescence [194].

Mechanical-based biosensors are generally composed of microcantilever systems based on different sensing principles: stress detection or mass detection. For the stress detection mode, biochemical interactions between pathogens and the sensitized surface of a cantilever result in the alteration of surface-free energy and surface stress on both sides. Consequently, the mechanical deflection of the cantilever can be measured along with the label-free detection of the bioanalyte [204]. For the mass detection mode, a biosensor equipped with a piezoelectric surface immobilized with antibodies is placed in a solution containing pathogens. Then an increase of the crystal mass is generated because of the attachment of the agent to the antibody coated surface, resulting in a measurable corresponding resonance frequency shift [197]. This detection method generally includes three types of applications: quartz crystal microbalance (QCM), surface acoustic wave (SAW) and magnetoelastic detection [210-212].

Implantable medical devices, some of which are BioMEMS-based systems, have been used to restore lost or damaged organ functions in the body, benefiting many people and improving their quality of life. The most important considerations concerning the implantation are safety and the reliable performance of the devices in their designed lifetime. Furthermore, the implanted devices have to be compatible with the mechanical properties of the organ, tissue and the microenvironment of the cells within [171,198]. In some cases, the systems stay inside the body for years and some even stay for a life time. Therefore, biocompatibility is always at the top of the list of critical requirements. Neural prostheses are an important application of BioMEMS devices [199,200]. Using electrical pulses to simulate intercellular communication can help restore the lost neural activity. For example, retinal implants are designed to restore sight [201,202] and cochlear implants are designed to restore hearing [203,204]. There are two types of retinal implants, epiretinal implants and subretinal implants. The selection is based on the condition of the patient. If the patient's photoreceptors are not functional, then a subretinal implant is selected, otherwise, an epiretinal implant can be used [201,202]. If the optic nerve is damaged or degenerated, retinal implants are no longer an option and, in this case, brain implants are utilized through the occipital lobe, which is responsible for processing visual signals. Compared to retinal implants, this approach is more challenging due to the complex organization of the nerve cells and the mechanical properties of the soft brain tissue. There have been significant research efforts to design and develop the next generation of implantable BioMEMs devices, which embody complex functionalities and better adapt to the physical environment of the host tissue or organ [205-207].

### 5.5 Neuroscience

Neurons are widely regarded as one of the most complex cell types within the body. Their unique anatomy strongly influences their electrochemical function of signal propagation and transmission.

The shape of an axon greatly affects signal conductivity spatially or temporally. As such, the physical geometry of a chip on which neurons are interfaced is of critical importance. Another consideration in basic design is the electrode; it is an essential tool that can both measure neuronal performance and apply stimuli. Since both platform geometry and electrodes are generally the most critical aspects in neuroscience research, the primary driving factor in the choice of topography and electrode type of BioMEMS is the type of research being performed.

The actual physical geometry of the micro-scale platform remains the most crucial feature for the development and research of BioMEMS neurons. Neurons are commonly segregated from each other and isolated into individual chambers. The design of these small compartments allows for the direct confinement of neuronal somas, axons and dendritic branches in separate chambers connected by a series of micro tunnels [208-210]. By using this basic confinement technique, a wide variety of research options becomes possible, as specific structures can be isolated and investigated.

Although electrodes have diversified into many different shapes and materials in order to fulfil different roles, their primary functions of stimulation and recording remain the same. A common two-dimensional multi-electrode array consists of a configuration of flat contacts on which neurons are cultivated or patterned [211]. An example of this is demonstrated with neural progenitor cells that are patterned directly onto a multielectrode array (MEA) in order to monitor elicited bursting activity [212]. (Other types of MEAs, such as needle arrays, will be mentioned later).

Developments in optogenetics have allowed for the creation of genetically modified neurons that experience fluoresce when activated or that can be stimulated using light. Recent developments in single-cell fluorescent manipulation have resulted in the creation of a probe with a microscopic tip that is resolute enough for individual cells [213]. The optical fibre can detect individual fluorescing neurons with high reliability *in vivo*; it can be used to optically stimulate an individual cell without affecting the neighbouring population [213].

Guiding axons in a specific direction using compartmentalization techniques can yield interesting research possibilities. A common technique in BioMEMS research is the culturing of neurons such that they produce rows of axons in parallel microchannels. Other methods for guiding axon propagation involve utilizing techniques such as soft lithography [214]; this process can be used for “inking” proteins or growth factors attracting or repelling the neurites, thus effecting their growth direction. Under certain conditions, axon growth can be polarized into specific directions, allowing for construction of neural circuits [215]. Compartmentalized culturing plays a key role in neuronal co-cultures. Using micro-scale platforms to create small cultures of neurons alongside other bodily cells allows for research into interactions between neurons and other cells. Investigations into behaviour can involve, for instance, circuitry originating from a central nervous system neuron to that of the peripheral nervous system, and ending on a myocyte, replicating a miniature signal pathway from the brain to a muscle [208].

Co-cultures of different types of cells are useful when attempting to mimic the environment *in vivo.* Takeuchi et al. [216] co-cultured rodent superior cervical ganglion neurons and ventricular myocytes, and then seeded them together; neural connections were made and the electrical activity monitored.

Areas of research that focus on axon manipulation frequently involve disease models where a neuron has sustained some sort of physical damage. Axotomy procedures are used to simulate the severing of the axon, something which could otherwise occur to victims of traumatic accidents. Methodologies developed for inflicting damage to the axon may involve processes such as laser transection, where a precision laser beam severs the axon [217]. Another method for axotomy is using vacuum aspiration, when a pinpoint vacuum is applied to an extended region of the axon in order to destroy that section [218]. Yet another methodology that has been demonstrated involved a miniature device that pinched the axon at variable forces to simulate compressive damage [219]. Once the axon has been damaged, the dynamics of the neuron can be studied in order to see how the cell responds to the trauma.

One attribute commonly associated with the development of Alzheimer's disease is the buildup of insoluble beta amyloids that occur as a result of amyloid protein cleaving. These beta amyloids plaque themselves between synaptic connections in the brain and inhibit signals between neurons. BioMEMS platforms were used to demonstrate how the beta amyloid proteins interfere with neurotrophin growth factors, a protein integral to neuron function [220]. Typical cultures in petri dishes produce unorganized tangles of neural fibres that normally hinder the results of these kinds of experiments, but with a BioMEMS neuron culture with a propagated parallel axon, beta amyloids can easily be introduced to the isolated synaptic branches.

### 5.6 Neurophysiology

Some of the most fundamental questions in the field of neuroscience concern how physiological and behavioural functions are controlled by neuronal circuits, and how these circuits affect each other. To answer these questions, several types of electrophysiological techniques have been developed over the past few decades, such as intracellular recordings with sharp or patch electrodes, or extracellular recordings with single electrodes or stereotrodes. These techniques can provide us with information regarding neural cell function *in vivo* and *in vitro*, and are capable of recording the activities of a single cell or several neurons at a time. Unfortunately, these existing methods are not capable of monitoring the complete repertoire of biophysical properties of each cell in a circuit, because of physical and electrical limitations, and the devices' relatively low throughput. It is becoming increasingly evident that in order to map the brain's synaptic characteristics at the micro- and nanoscale, the recording device also needs to be scaled accordingly. In the past decade there have been many attempts to miniaturize and further improve the existing recording tools. As part of this process, many laboratories introduced techniques that attempt to combine the advantages of extracellular electrode arrays with the benefits of intracellular electrodes creating the new era of micro-scale MEA.

**Figure 4.**
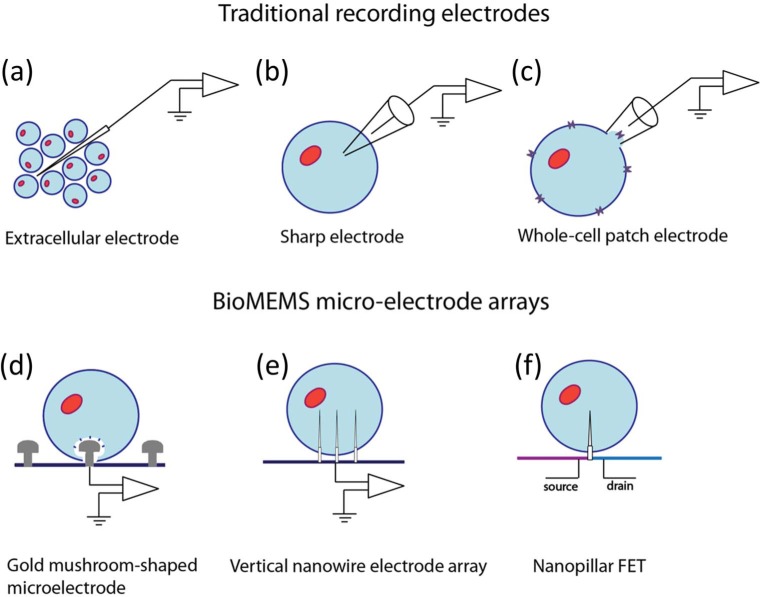
Different forms of electrophysiological recording techniques. Shown above are schematic illustrations of traditional electrodeneuron interface configurations and BioMEMS microelectrode arrays. In the schematics, neurons are depicted in light blue (somas are marked with orange) (a) Extracellular recording electrode. The electrode does not penetrate any of the cells, thus it can record the activity of multiple neurons. (b) Intracellular recording with a sharp glass microelectrode. (c) Whole-cell patch clamp technique. This technique allows us to study single or multiple ion channels (marked with purple) located on a membrane patch of a single cell. (d) Gold mushroom-shaped microelectrodes are actively engulfed by neurons because of their dendritic spine-like shapes. The mushroom-shaped protrusion is 1.42μm high. (e) A vertical nanowire electrode array (VNEA) that penetrates the cell membrane providing direct contact with the cell. (f) A pillar-shaped protruding nanowire is the sensing gate electrode of the FET.

In a network of cells, the neuron sending the action potential is called the presynaptic cell and the one receiving it is called the postsynaptic cell. Relationships between neurons can be inhibitory and excitatory depending on the type of neurotransmitter released by the cells [3]. Action potentials can be recorded with both extra- and intracellular techniques (Figure 4a-c), however, the readouts look different depending on the applied technique. When designing a MEA, it is critical that the device has the capability to detect supra- and subthreshold membrane potentials as well. Intracellular recording techniques, such as the ones illustrated by Figure 4b&c, can monitor changes in voltage in the membrane potentials, as well as the current changes caused by the ion flow in the cells, which is one of the main reasons why MEAs need to be able to penetrate the cytosol of the neurons.

By utilizing extracellular techniques (Figure 4a) investigators can also monitor the synchronized activity of large ensembles of neurons. This type of additive signal is called local field potential (LFP). In this case, voltage is generated as the sum of the current flow in the population of local neurons [221]. Based on the LFPs recorded, investigators can analyze the network level changes in activity in the brain more generally. These oscillations combined with the intracellular properties and potentials of single neurons can carry substantial information regarding the network level and single cell modulations that can be highly meaningful when characterizing a brain region's connectivity.

As mentioned previously, an ideal MEA needs to utilize the advantages of intracellular and extracellular recording systems, and do so in a high throughput manner in order to maximize the amount of information gained from a recording. These arrays need to have great electrical coupling with each single cell they are recording from, like glass sharp and patch electrodes do, but without having to worry about the short duration of the recording due to mechanical or biophysical instabilities. Therefore, they need to be flexible enough that small distortions will not cause them to break, but stiff enough that they can keep a recording stable for longer periods of time (days or even months). An ideal device needs to be able to record the relevant transmembrane potentials, action potentials, EPSPs and IPSPs, as well as LFPs [222].

Since 2007, Spira et al. have been working on creating a new type of MEA that has an improved cell adhesion property compared to conventional recording techniques [223-227]. As a result of this project, they were able to create an electrode design that is capable of establishing not only chemical, but also biological adhesion with the cell. As illustrated by Figure 4d, the electrodes on the MEA are specifically shaped to a micrometre sized gold mushroom protrusion that is covered with a chemical attractant to create a tight connection with the neuron [227]. This camouflaged appearance contributes to a higher electrical coupling because the cells tend to actively engulf the electrode by endocytosis. The MEA when tested in Aplysia neuron culture showed no significant changes in input resistance before and after the recording, and stimulation session, thus the array can be used to stimulate the cells to fire action potentials without causing any damage to them. The array was capable of recording action potentials of up to 25 mV intracellularly from multiple cells over two days due to the stable cell-electrode coupling [227], which has great potential for application to *in vivo* chronic recordings.

A different approach was taken when Robinson et al. fabricated electrodes utilizing nanopillars for intracellular recordings [228-230]. In this study they created vertical nanowire electrode arrays (VNEAs) that allowed parallel electrical connections with ensembles of rat cortical neurons. The schematic illustration of the VNEAs is shown in Figure 4e. The VNEA's planar geometry makes it a great candidate for high throughput *in vitro* recordings, as well as recordings performed in slice preparations; however, it is not ideal for *in vivo* preparations. When tested in rat cortical neurons, approximately half of the electrode tips penetrated the cells spontaneously. The other 50% successfully penetrated after a short electroporating current was applied to the cells, which is one of the major design flaws. It has been shown that recordings following electroporation are only transient, because the current activates cell repair mechanisms that close up the membrane and consequently make the electrode extrude[225]. Despite this problem, the array provides a promising new method that is capable of short-term, multi-site, single cell, high-throughput, intracellular recordings [222,230].

## 6. Future directions

Cell mechanics as a research area is a crucial discipline that bridges cell biology and biochemistry with the help of micro and nano-engineered technologies. Experimental and computational mechanics provide a detailed understanding about essential connections among structures, mechanical properties and functions of cells. Although many efforts in cell mechanics have already been aimed towards understanding how cells move, sense, deform and interact with their microenvironment, we must continue to study how the mechanical properties of cells change during a state of disease and how these changes impact signaling processes. Despite remarkable progress to date, there are still many opportunities as yet unexplored to study cell mechanics in reproduction, tissue repair as well as in a long list of diseases. BioMEMS devices open new venues in studying mechanical aspects of cells due to their cost-effective, relatively easy fabrication, and user-friendly nature. Even though BioMEMS devices have given biomedical researchers unprecedented capabilities in cell mechanics, there is still room for advancement and improvement, in characterizing and manipulating cells mechanically. An important direction is nano and micro-scale sensing technologies that can adapt to the 3D environment of cells.

We are still confronted with the challenges of exploring and understanding the mechanotransduction scheme, and metastatic mechanism of cancers. The solutions to these problems lie in quantifying the interplay between cancer cell mechanics and the underlying chemistry. Cancer cell mechanics is still a fledgling field, which requires a better understanding of the mechanotransduction scheme and the metastatic mechanism, and is inspiring more innovative techniques to translate those experimental results into clinical applications.

Some areas of neuroscience have also benefited from BioMEMS. These miniature systems provide us with an improved ability to physically manipulate neurons, and initiate precision signal stimulation and recording. As we attempt to more closely mimic *in vivo* human body environments, it is likely that we will see more complex neural patterning, accurate three-dimensional cultures, better manipulation of neuronal behaviour, more efficient neural-electrode interfaces, and an increase in the number of implantable devices.

From a more general perspective, today's BioMEMS technologies are basically building small channels or simple solid structures, and most of these tools rely on the simplest mechanical laws. To put this in an analogy, the tools that we have so far are similar to primitive mechanical tools that people were using during the middle ages. Since the renaissance, with advancements in power generation, electrical and information breakthroughs, we have had versatile, automated, durable mechanisms in almost all of the tools we use every day. For the future of BioMEMS devices in biomedical areas, the incorporation of mechanical, electrical, optical and information technologies has great potential for developing efficient, versatile and comprehensive analysis tools. A single microengineered device that could measure multiple quantities in a short amount of time would be the ultimate quest of BioMEMS devices.
